# Supplementary figures and images for: Understanding the Polymerization of Polyfurfuryl Alcohol: Ring Opening and Diels-Alder Reactions
Source: Polymers (Basel). 2019 Dec 17;11(12):2126. doi: 10.3390/polym11122126 (PMC6969920; doi:10.3390/polym11122126)

— PFA  
— FA

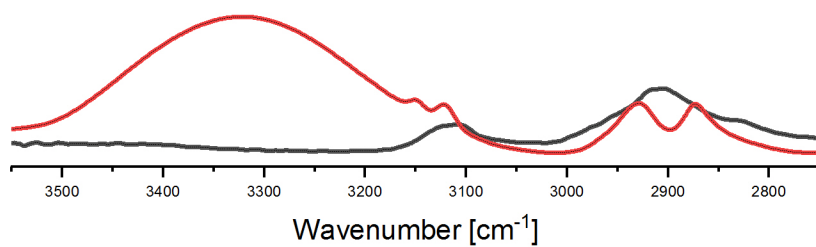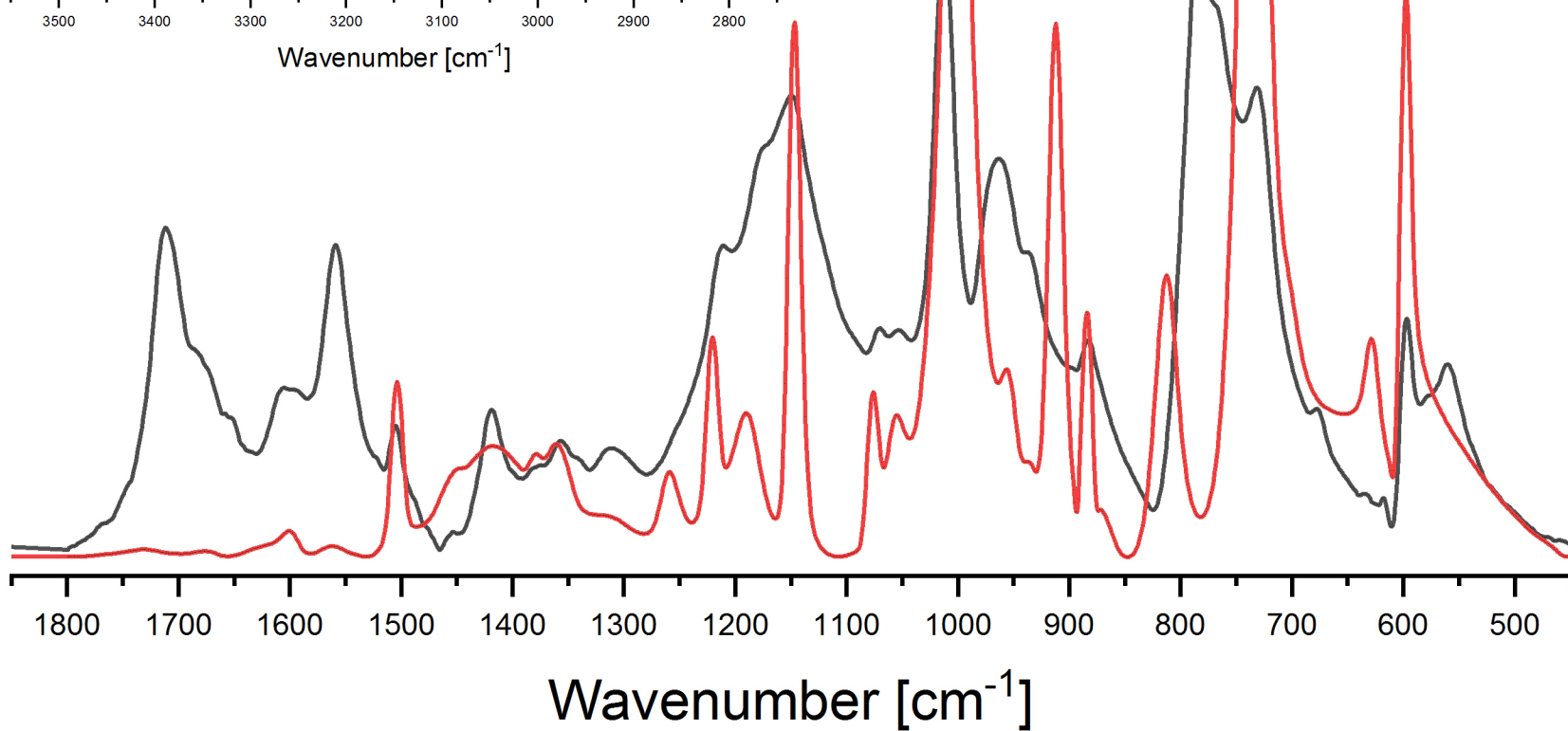

Supplement: Supplementary file 1 [file polymers-11-02126-s001.pdf]
